# ANGPTL4 Expression Is Increased in Epicardial Adipose Tissue of Patients with Coronary Artery Disease

**DOI:** 10.3390/jcm11092449

**Published:** 2022-04-27

**Authors:** Yasufumi Katanasaka, Ayumi Saito, Yoichi Sunagawa, Nurmila Sari, Masafumi Funamoto, Satoshi Shimizu, Kana Shimizu, Takehide Akimoto, Chikara Ueki, Mitsuru Kitano, Koji Hasegawa, Genichi Sakaguchi, Tatsuya Morimoto

**Affiliations:** 1 Division of Molecular Medicine, School of Pharmaceutical Sciences, University of Shizuoka, 52-1 Yada, Suruga-ku, Shizuoka 422-8526, Japan; katana@u-shizuoka-ken.ac.jp (Y.K.); chopin.nocturne.op.152@gmail.com (A.S.); y.sunagawa@u-shizuoka-ken.ac.jp (Y.S.); nurmilasari@gmail.com (N.S.); funamoto@tokushima-u.ac.jp (M.F.); s18410@u-shizuoka-ken.ac.jp (S.S.); s18804@u-shizuoka-ken.ac.jp (K.S.); koj@kuhp.kyoto-u.ac.jp (K.H.); 2 Division of Translational Research, National Hospital Organization Kyoto Medical Center, 1-1 Mukaihata-cho Fukakusa, Fushimi-ku, Kyoto 612-8555, Japan; 3 Laboratory of Clinical Cardiovascular Pharmacology, Shizuoka General Hospital, 4-27-1 Kita Ando Aoi-ku, Shizuoka 420-8527, Japan; 4 Department of Cardiovascular Surgery, Shizuoka General Hospital, 4-27-1 Kita Ando Aoi-ku, Shizuoka 420-8527, Japan; takeakimoto@yahoo.co.jp (T.A.); uekichikara@gmail.com (C.U.); katanakun2002@yahoo.co.jp (M.K.); 5 Department of Cardiovascular Surgery, Kindai University Faculty of Medicine, 377-2 Ohno-Higashi, Osaka-Sayama 589-8511, Japan; genichisakaguchi@gmail.com

**Keywords:** coronary artery disease, epicardial adipose tissue, angiopoietin-like 4, interleukin-1β

## Abstract

Epicardial adipose tissue (EAT) is known to affect atherosclerosis and coronary artery disease (CAD) pathogenesis, persistently releasing pro-inflammatory adipokines that affect the myocardium and coronary arteries. Angiopoietin-like 4 (ANGPTL4) is a protein secreted from adipose tissue and plays a critical role in the progression of atherosclerosis. Here, the expression of ANGPTL4 in EAT was investigated in CAD subjects. Thirty-four consecutive patients (13 patients with significant CAD; 21 patients without CAD) undergoing elective open-heart surgery were recruited. EAT and pericardial fluid were obtained at the time of surgery. mRNA expression and ANGPTL4 and IL-1β levels were evaluated by qRT-PCR and ELISA. The expression of ANGPTL4 (*p* = 0.0180) and IL-1β (*p* < 0.0001) in EAT significantly increased in the CAD group compared to that in the non-CAD group and positively correlated (*p* = 0.004). Multiple regression analysis indicated that CAD is a contributing factor for ANGPTL4 expression in EAT. IL-1β level in the pericardial fluid was significantly increased in patients with CAD (*p* = 0.020). Moreover, the expression of ANGPTL4 (*p* = 0.004) and IL-1β (*p* < 0.001) in EAT was significantly increased in non-obese patients with CAD. In summary, ANGPTL4 expression in EAT was increased in CAD patients.

## 1. Introduction

Adipose tissue is recognized to contribute to physiological regulation and pathological processes, such as metabolic syndromes and inflammatory disorders [1]. It is an endocrine organ that secretes multiple adipokines and inflammatory cytokines that exert local and systemic effects on the heart and vasculature. These secreted proteins may alter the functions of endothelial cells, arterial smooth muscle cells, and inflammatory cells, leading to the development of atherosclerosis and coronary artery disease (CAD) [2].

Interest in organ-specific adipose tissue is rapidly growing as a substantial accumulation of scientific evidence indicates that anatomic specificity is an important contributor to the pathophysiology of various metabolic diseases. In this context, epicardial adipose tissue (EAT), which is the visceral fat of the heart, has emerged as an adipose tissue of interest in the study of cardiometabolic diseases. Some clinical studies have shown that EAT volume is associated with the risk of CAD events [3]. In animal models, the excision of EAT has been shown to decrease coronary atherosclerosis [4]. Furthermore, the transcriptome of epicardial fat is markedly different from that of subcutaneous fat, with the majority of relatively enriched genes being associated with endothelial function, coagulation, and inflammation [5]. These reports suggest that EAT is involved in atherosclerosis and CAD development by producing multiple cytokines locally in the atherosclerotic region; however, such a process has not yet been elucidated.

Angiopoietin-like 4 (ANGPTL4) is a multifunctional protein secreted from adipose tissue and liver [6]. Its expression is upregulated by fasting, hypoxia, and obesity [7]. It is known to be an inhibitor of lipoprotein lipase (LPL) and increases plasma triglyceride (TG) levels systemically [8]. It has been reported that ANGPTL4 knockout mice exhibit increased peripheral LPL activity and decreased TG levels [9]. Consistent with the findings in mice, cynomolgus monkeys and hyperlipidemic rhesus monkeys treated with an ANGPTL4-neutralizing monoclonal antibody decreased the levels of plasma TG [10]. Two large clinical studies indicate that a loss-of-function mutation (E40K) in ANGPTL4 is associated with reduced plasma TG and risk of CAD [10,11]. These studies indicate that ANGPTL4 secreted in pathological conditions may worsen lipid metabolism and atherosclerosis. Although ANGPTL4 is also expressed in EAT, the change in the expression in patients with or without CAD and the local effect of ANGPTL4 in the atherosclerotic region has not been clarified.

Here we hypothesized that ANGPTL4 expression in EAT is involved in the development of CAD. Dysregulated secretion of adipocytokines appears to trigger obesity-associated chronic inflammation and contributes to the development of cardiovascular atherosclerosis [12]. Pro-inflammatory cytokines and adipokines have been shown to be expressed at a higher level in the adipose tissue of patients with CAD than in those without CAD [13,14]. In this study, we investigated the expression of ANGPTL4 in the EAT of patients with or without CAD and its association with inflammatory cytokines, such as interleukin 1-beta (IL-1β), IL-6, and tumor necrosis factor-α (TNF-α).

## 2. Materials and Methods

### 2.1. Patients

This study was conducted in accordance with the Declaration of Helsinki, and the protocol was approved by the Ethics Committee of Shizuoka General Hospital (Project identification code: 12-03-56), conducted between November 2012 and January 2013. Samples were obtained from 13 patients who underwent coronary artery bypass graft surgery (CAD group) and 21 patients who underwent open-heart surgery (non-CAD group) at Shizuoka General Hospital. Among 13 CAD subjects, 10 unstable angina, 2 exercise-induced angina, and 1 myocardial infarction were included. Among 21 non-CAD subjects, 12 aortic or mitral valve diseases, 7 aortic aneurysm, and 1 cardiac tumor were included. Written informed consent was obtained from each patient. EAT was excised from the anterior wall of the left ventricle. Individuals deemed unsuitable for this trial by their primary physician were excluded.

### 2.2. Blood Tests

Blood samples were obtained in tubes without anticoagulant or with EDTA sodium. Plasma and serum were separated by centrifugation according to the protocol of Shizuoka General Hospital. Pericardial fluids were separated by centrifugation at 3000 rpm at 4 °C for 10 min to remove the debris. The following clinical data were collected by chart review: aspartate aminotransferase (AST), alanine aminotransferase (ALT), estimated glomerular filtration rate (eGFR), total protein (TP), insulin, low-density lipoprotein cholesterol (LDL-C), high-density lipoprotein cholesterol (HDL-C), and triglyceride (TG). The levels of plasma glucose and brain natriuretic peptide (BNP) were also measured. White blood cell count (WBC), hemoglobin (Hb), and platelet count (PLT) were measured in whole blood.

### 2.3. qRT-PCR

Total RNA was isolated, reverse-transcribed, and amplified, as previously described, with some modifications [15]. Total RNA from the EAT was isolated using the RNeasy Lipid Tissue Mini kit (QIAGEN, Tokyo, Japan) and subjected to reverse transcription using ReverTra Ace qPCR RT Master Mix (Toyobo, Osaka, Japan). Quantification of RNA was performed using a LightCycler 96 Real-Time PCR System (Roche, Tokyo, Japan) using KOD SYBR qPCR Mix (Toyobo, Osaka, Japan). The primer sequences used in this study are shown in Table 1. The relative gene expression values were calculated using the ΔΔCt method (normalized to 18S rRNA).

### 2.4. ELISA

Pericardial fluids obtained from the patients, as described above, were used in the ELISA assay. Samples were stored at −80 °C until the analysis. The amounts of ANGPTL4 and IL-1β from the patients were measured with quantitative colorimetric sandwich ELISAs (R&D Systems, Minneapolis, MN, USA and Proteintech, Tokyo, Japan) as described previously [6,16]. For all ELISAs, protein concentrations were calculated using a standard curve generated with recombinant standards provided by the manufacturer. Optical density was measured using a microtiter plate reader at 450 nm. Each sample was measured in duplicate and averaged.

### 2.5. Statistical Analysis

Data are expressed as the mean ± standard deviation (SD). Statistical comparisons were performed using the Student’s t-test or Fisher’s exact test. Correlations were assessed using Spearman’s rank correlation for non-normally distributed data. Multivariate analysis was performed by the adjustment for age, gender, established risk factors (hyperlipidemia and diabetes mellitus); *p* < 0.05 indicated significance. Statistical analyses were performed using JMP version 12 software (SAS Institute Inc., Cary, NC, USA) or GraphPad Prism version 9 software (GRAPH PAD software Inc., San Diego, CA, USA).

## 3. Results

### 3.1. Patient Background

Thirty-four patients aged 56–84 years were enrolled in this study after providing written informed consent. The demographic characteristics and dispositions of the patients are shown in Table 2. Non-CAD patients (*n* = 21) had heart disease, including valve heart disease (*n* = 13), aortic aneurysm (*n* = 7), and pericardial tumors (*n* = 1), and underwent surgery as treatment. These subjects were administered various medications to treat their underlying diseases. The frequency of medicine with ARBs (*p* = 0.005) and statins (*p* = 0.004) was significantly higher in CAD patients than in non-CAD patients. Basic characteristics, including sex, age, and body mass index (BMI), were not significantly different between patients with non-CAD and CAD. There was no significant difference between these groups in smoking and drinking alcohol histories.

### 3.2. Clinical Evaluation

As shown in Table 3, the values of RBC (*p* = 0.011), PPG (*p* = 0.041), and HbA1c (*p* = 0.010) were significantly higher in patients with CAD than in those without CAD. The biomarkers associated with liver and kidney functions were not significantly different between patients with and without CAD.

### 3.3. ANGPTL4 Expression in EAT Was Increased in the Patients with CAD

The metabolic activity of EAT can be a major contributor to the disruption of physiological regulation in atherosclerosis. In fact, epicardial fat is the source of a number of bioactive cytokines that can either protect or adversely affect the myocardium and coronary arteries [17]. Since ANGPTL2, 3, and 4 are associated with lipid metabolism and atherosclerosis [18], we examined the expression of ANGPTL2, 3, 4, and inflammatory cytokines in the EAT of patients with CAD and non-CAD by qRT-PCR.

**Figure 1:**
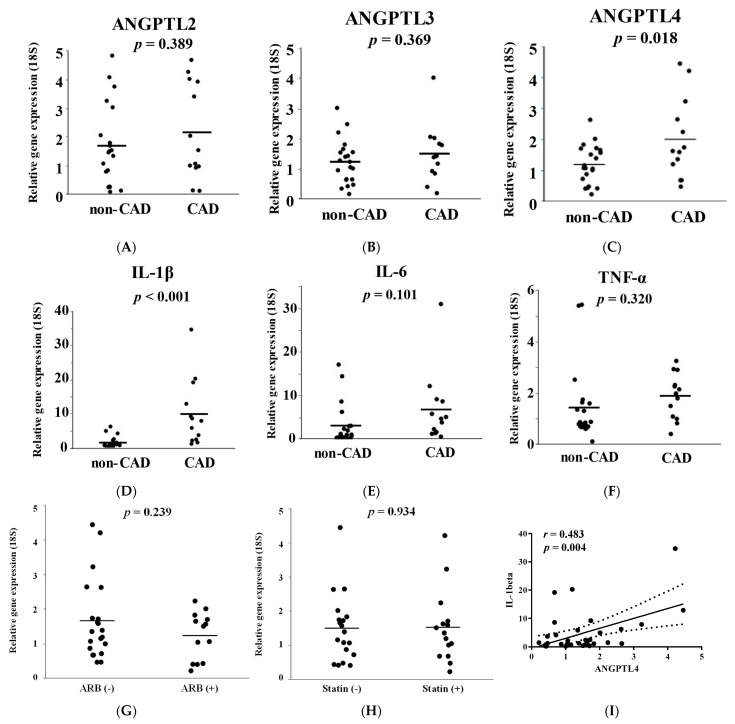
Angiopoietin-like 4 (ANGPTL4) and interleukin 1-beta (IL-1β) expression is increased in the epicardial adipose tissue (EAT) of patients with coronary artery disease (CAD). (**A**–**F**) Comparison of the gene expression of ANGPTL and inflammatory cytokines in EAT in patients with or without CAD. Two-tailed unpaired Student’s t-tests were used to compare non-CAD and CAD subjects. (**G**,**H**) Comparison of ANGPTL4 mRNA expression in the EAT of patients with or without ARBs (**G**) and statins (**H**). (**I**) Linear regression analysis was performed in the combined groups of non-CAD and CAD subjects. R and *p*-values are shown in the Figure.

The expression of ANGPTL2 and 3 were not significantly different between these groups (Figure 1A,B). ANGPTL4 expression was significantly increased in the EAT of CAD patients compared to that in the EAT of non-CAD patients (*p* = 0.018, Figure 1C). Pro-inflammatory cytokines, such as IL-1β, IL-6, and TNF-α play important roles in atherosclerosis progression and are associated with adipokines. In this study, we found that the expression of IL-1β in EAT was significantly higher in CAD patients than in non-CAD patients (*p* = 0.0005, Figure 1D–F). As shown in Table 2, ARB and statin medications were used more preferentially in CAD patients than in non-CAD patients. We examined the effect of these medications on ANGPTL4 expression in EAT. There was no significant difference in ANGPTL4 expression in EAT between the patients treated with statins or ARBs (Figure 1G,H), which suggested that these medications were not associated with changes in the expression of ANGPTL4. Although the number of patients with diabetes in the CAD group was higher than that in the non-CAD group, ANGPTL4 expression in EAT was not significantly altered in patients with or without diabetes. To clarify the association between ANGPTL4 and IL-1β, we investigated the correlation between ANGPTL4 and IL-1β in each patient. The results showed that the expression of ANGPTL4 was correlated with that of IL-1β in EAT (*p* = 0.004, *r* = 0.483, Figure 1I). Multiple regression analysis was performed on relationships to ANGPTL4 expression of combinations of possible determinants of age, gender, and clinical risk factors (diabetes mellitus and hyperlipidemia) (Table 4). The results also suggest that CAD is a contributing factor for ANGPTL4 expression in EAT.

### 3.4. IL-1β Secretion in Pericardial Fluid Was Increased in the Patients with CAD

**Figure 2:**
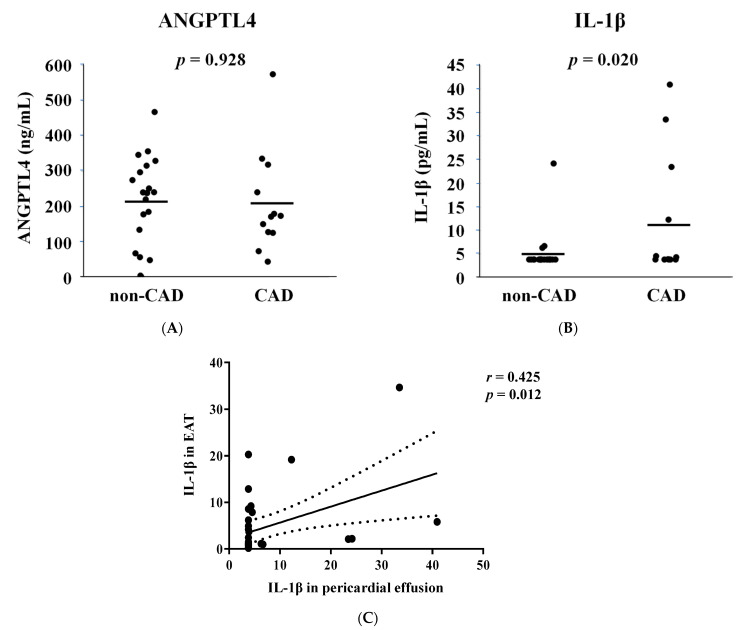
Interleukin 1-beta (IL-1β) secretion into the pericardial fluid is increased in patients with CAD. (**A**,**B**) Comparison of the protein concentration of angiopoietin-like 4 (ANGPTL4) and IL-1β in the pericardial fluid of patients with or without coronary artery disease (CAD). Two-tailed unpaired Student’s t-test was made between non-CAD and CAD subjects. (**C**) Linear regression analysis was performed in a combined group, including non-CAD and CAD subjects. R and *p* values are shown in the Figure.

Pericardial fluid contains various secreted bioactive factors, including atrial and brain natriuretic peptides and endothelin-1 [19]. Some of these active proteins are present at higher levels in pericardial fluid than in peripheral blood [19]. Moreover, it has been reported that EAT is involved in the adverse effects of chronic inflammation on coronary atherosclerosis [20]. Thus, we evaluated ANGPTL4 and IL-1β concentrations in the pericardial fluid of patients. ANGPTL4 protein levels in pericardial fluid did not increase in patients with CAD (Figure 2A,B). However, IL-1β concentration was significantly higher in CAD patients than in non-CAD patients (Figure 2C). IL-1β concentration in the pericardial fluid correlated with its expression in the EAT (r = 0.425, *p* = 0.012, Figure 2C).

### 3.5. ANGPTL4 and IL-1β Expression in EAT Was Increased in Non-Obese CAD Patients

**Figure 3:**
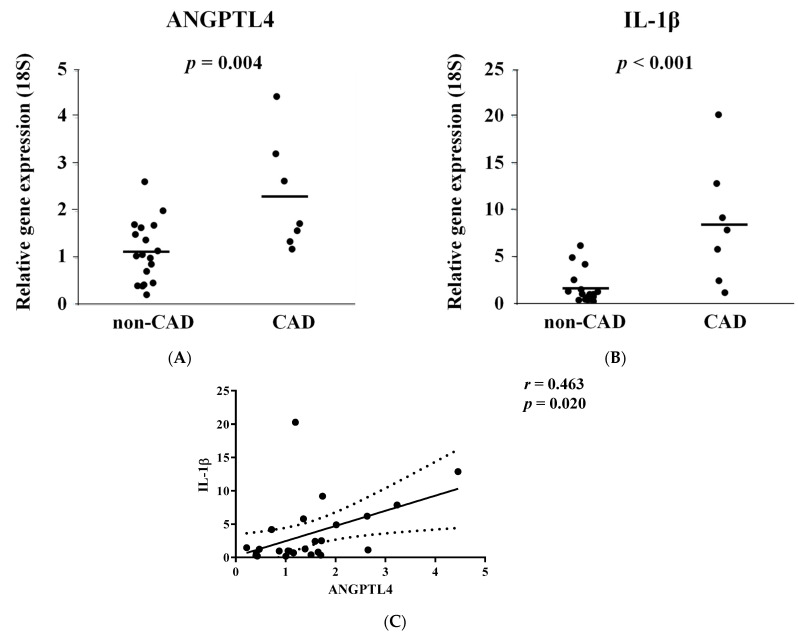
Angiopoietin-like 4 (ANGPTL4) and interleukin 1-beta (IL-1β) expression is increased in epicardial adipose tissue (EAT) of the non-obese patients with coronary artery disease (CAD). (**A**,**B**) Comparison of the gene expression of ANGPTL4 and IL-1β mRNA in EAT in non-obese patients (BMI < 25) with or without CAD. Two-tailed unpaired Student’s t-test was made between non-CAD and CAD subjects. (**C**) Linear regression analysis was performed in a combined group, including non-CAD and CAD subjects. R and *p* values are shown in the figure.

Obesity is a significant risk factor for atherosclerosis and CAD onset. Additionally, the critical risk factors in non-obese patients have been under investigation. The accumulation of EAT is associated with CAD development in non-obese patients [21]. We performed stratification analysis on the expression of ANGPTL4 and IL-1β in non-obese patients (cut-off: BMI < 25) and found that ANGPTL4 and IL-1β were significantly upregulated in EAT of non-obese patients with CAD compared to that in non-CAD patients (Figure 3A,B). Additionally, the levels of expression were significantly correlated (Figure 3C).

## 4. Discussion

In this study, ANGPTL4 mRNA expression was found to increase in the EAT of CAD patients compared to that of non-CAD patients, while IL-1β increased in the pericardial fluid of CAD patients. Inflammatory cytokines such as IL-1β are involved in atherosclerosis progression. These data suggest that ANGPTL4 upregulation in EAT and IL-1β expression promote atherosclerosis locally in the pathological region of the coronary artery.

Alterations in ectopic lipid deposition and circulating lipids are considered independent risk factors for cardiometabolic disorders, including atherosclerosis [22,23]. Accumulating evidence indicates that ANGPTL4 is associated with TG metabolism and atherosclerosis progression [10,11,24,25]. Thus, previous studies have revealed the importance of ANGPTL4, which is known as a regulator of LPL in cardiometabolic disorders such as atherosclerosis. However, in our study, the plasma ANGPTL4 and TG levels were not changed between CAD and non-CAD patients (data not shown). ANGPLT4 is known to be a multiple functional protein [18]. The overexpression of ANGPTL4, specifically in adipose tissue, decreases LPL activity in adipose tissue and the plasma [26,27]. A recent study in mice showed that the loss of ANGPTL4 in adipose tissue decreases circulating TG and cholesterol levels and atherosclerosis. In addition to decreased circulating lipids, a lack of ANGPTL4 in adipocytes also decreases vascular inflammation and endothelial activation in the aorta of mice, suggesting a pro-inflammatory and proatherogenic role of ANGPTL4 [27]. On the other hand, a protective effect of ANGPTL4 against inflammation and atherosclerosis has been reported [28,29,30,31]. The cell- and condition-specific functions of ANGPTL4 have been clarified in further studies.

We found that in EAT, IL-1β was correlated with ANGPTL4. The CANTOS trial suggested that the inhibition of IL-1β with canakinumab reduces cardiovascular events [32,33,34]. Several basic studies have revealed the effect of anti-inflammatory therapy targeting IL-1β on atherosclerosis, as well as its mechanism [35,36,37]. These studies have established the importance of IL-1β-associated inflammation in atherosclerosis and CAD. Aryal et al. showed that loss of Angptl4, specifically in adipose tissue, attenuates atherosclerosis in mice fed a western diet by reducing plasma inflammatory cytokines, including IL-1β [27]. ANGPTL4 has been shown to positively regulate NF-kB signaling, one of the most important transcription factors for inflammatory cytokines [38]. These results support our hypothesis that ANGPTL4 may be involved in IL-1β production in EAT. Atherosclerosis is a chronic inflammatory disease in the arterial subendothelial space [39]. Inflammation in the pericardial adipose tissue correlates with coronary artery disease and calcific aortic stenosis [40,41]. It has been reported that the pericardial fat volume was significantly associated with IL-6 concentration [42]. EAT has been reported to be a source of pro-inflammatory mediators [41] and the polarization of a pro-inflammatory macrophage subtype is increased in EAT of patients with heart failure [43]. These findings suggest that the inflammation status in EAT is altered by various pathological conditions and may contribute to the development and progression of disorders. It appears that ANGPTL4 can be one of the transducers of inflammation in EAT, but future studies will be needed to demonstrate the function of ANGPTL4 in inflammation in epicardial adipocytes.

An abundance of evidence supports the involvement of EAT in cardiovascular risk profiles of obese patients [44,45]. In addition, the accumulation of EAT is associated with CAD development in non-obese patients [21]. Previous reports have indicated that EAT volume is associated with CAD incidence independently of BMI, suggesting that the roles of EAT in non-obese patients may be different from those in obese patients [46,47]. In this context, we excluded obese patients (BMI ≥ 25) and analyzed ANGPTL4 and IL-1β expression in EAT in non-obese patients with CAD. Interestingly, ANGPTL4 and IL-1β expression in EAT was also found to be significantly increased in non-obese patients with CAD compared to those without CAD. Although the regulatory mechanism for this expression is unknown, ANGPTL4 and IL-1β may contribute to the development of atherosclerosis via the induction of local inflammation around EAT in individuals without obesity.

The present study has several limitations. The study design was cross-sectional, and the results did not imply causality for several reasons. First, computer tomographic data of the EAT showing the volume were lacking. Second, the number of samples was small for conclusive evidence. Third, there may have been an experimental bias present in patient selection. Fourth, the sample size is insufficient for adequate subgroup analysis. Fifth, the consequence of gene expression between ANGPTL4 and IL-1β in EAT is not determined.

## 5. Conclusions

In conclusion, we found for the first time that ANGPTL4 expression was increased in the EAT of patients with CAD, and its expression was positively correlated with that of IL-1β. Our findings suggest that ANGPTL4 in EAT can be a key mediator in controlling inflammation in atherosclerotic regions and might be a biomarker for CAD. This result may significantly help in understanding the mechanisms by which EAT metabolism accelerates atherosclerosis and CAD development. Further basic and clinical studies are needed to elucidate the relationship between adipokines secreted from EAT and the progression of atherosclerosis.

## Figures and Tables

**Table 1:** Primer sequences used in this study.

Target	Forward	Reverse
ANGPTL2	5′-ACGTACAAGCAAGGGTTTGG-3′	5′-ACGTACAAGCAAGGGTTTGG-3′
ANGPTL3	5′-ATTTTAGCCAATGGCCTCCT-3′	5′-ATTTTAGCCAATGGCCTCCT-3′
ANGPTL4	5′-TCCAGCAACTCTTCCACAAG-3′	5′-TCCAGCAACTCTTCCACAAG-3′
TNF-α	5′-CCTGTGAGGAGGACGAACAT-3′	5′-CCTGTGAGGAGGACGAACAT-3′
IL-1β	5′-TGAGCACCTTCTTTCCCTTC-3′	5′-TGAGCACCTTCTTTCCCTTC-3′
IL-6	5′-AGGCACTGGCAGAAAACAAC-3′	5′-AGGCACTGGCAGAAAACAAC-3′
18S rRNA	5′-CTTAGAGGGACAAGTGGCG-3′	5′-GGACATCTAAGGGCATCACA-3′

ANGPTL2-4: Angiopoietin-like 2-4, TNF-α: tumor necrosis factor-α, IL-1β: interleukin 1-beta, IL-6: interleukin 6, 18S: 18S ribosomal RNA.

**Table 2:** Demographic characteristics of subjects.

	Non-CAD	CAD	*p*-Value
Number	21	13	
Male	13 (62%)	10 (77%)	0.465
Ages (years)	72 ± 12	66 ± 10	0.170
BMI (kg/m^2^)	22.5 ± 3.3	23.7 ± 5.1	0.422
Complications			
Hypertension	11 (52%)	8 (62%)	0.728
Dyslipidemia	8 (38%)	9 (69%)	0.157
Diabetes	7 (33%)	8 (62%)	0.160
Chronic kidney disease	8 (38%)	7 (54%)	0.484
Cerebral ischemia	5 (24%)	4 (31%)	0.704
Atrial fibrillation	4 (19%)	1 (8%)	0.627
Medications			
ACE inhibitors	3 (14%)	4 (31%)	0.387
ARBs	12 (57%)	1 (8%)	**0.005**
Statins	5 (24%)	10 (77%)	**0.004**
Oral hypoglycemic agent	5 (24%)	4 (31%)	0.704
Insulin	1 (5%)	1 (8%)	1.000
Smoking history	12 (57%)	6 (46%)	0.725
Drinking history	7 (33%)	2 (15%)	0.427

Data are presented as means ± SD or number (%) of patients as indicated. *p* values were determined by Student t-test or Fisher exact test, with those less than 0.05 being highlighted in bold. BMI: body mass index, ACE: Angiotensin converting enzyme, ARB: angiotensin II receptor blocker.

**Table 3:** Clinical parameters of the patients.

	Non-CAD	CAD	*p* Value
SBP (mmHg)	122 ± 18	126 ± 24	0.581
DBP (mmHg)	70 ± 14	72 ± 17	0.804
EF (%)	63 ± 6	56 ± 15	0.054
E/A	0.83 ± 0.46	1.18 ± 0.76	0.148
E/E’	17.49 ± 9.71	14.17 ± 8.60	0.387
BNP (pg/mL)	289 ± 732	648 ± 1381	0.328
WBC (10^2^/µL)	50 ± 17	61 ± 15	0.057
RBC (10^4^/µL)	333 ± 42	377 ± 50	**0.011**
Plt (10^4^/µL)	17.7 ± 12.3	21.2 ± 4.8	0.332
Hb (g/dL)	10.2 ± 1.7	11.3 ± 1.8	0.079
Ht (%)	30.6 ± 4.6	33.7 ± 4.7	0.073
TG (mg/dL)	114 ± 86	108 ± 37	0.807
LDL-C (mg/dL)	88 ± 30	72 ± 20	0.111
HDL-C (mg/dL)	47 ± 14	41 ± 10	0.153
PPG (mg/dL)	120 ± 25	139 ± 26	**0.041**
HbA1c (NGSP %)	5.6 ± 0.7	6.6 ± 1.4	**0.010**
Insulin (µU/mL)	12.1 ± 11.5	10.9 ± 5.4	0.728
TP (g/dL)	6 ± 0.9	6.5 ± 0.8	0.092
Alb (g/dL)	3.2 ± 0.5	3.3 ± 0.6	0.413
AST (U/L)	20 ± 13	17 ± 8	0.447
ALT (U/L)	21 ± 35	17 ± 14	0.715
eGFR (mL/min)	53 ± 24	60 ± 25	0.425
CRP (mg/dL)	0.37 ± 0.86	1.53 ± 4.48	0.253

Data are presented as means ± SD or number (%) of patients as indicated. *p* values were determined by Student t-test, with those less than 0.05 being highlighted in bold. CAD: coronary artery disease, SBP: systolic blood pressure, DBP: diastolic blood pressure, EF: ejection fraction, E/A: the ratio of early filling (E) and atrial contraction (A) transmitral flow velocities, E/E’: the ratio of early diastolic mitral inflow velocity to early diastolic mitral annular velocity, BNP: brain natriuretic peptide, WBC: white blood cell count, RBC: red blood cell count, PLT: platelet count, Hb: hemoglobin, Ht: hematocrit, TG: triglyceride, LDL-C: low-density lipoprotein cholesterol, HDL-C: high-density lipoprotein cholesterol, PPG: postprandial glucose, TP: total protein, Alb: albumin, AST: aspartate aminotransferase, ALT: alanine aminotransferase, eGFR: estimated glomerular filtration rate, CRP: C-reactive protein.

**Table 4:** Multivariate analysis to estimate ANGPTL4 expression.

*R* ^2^	0.3367		
Corrected *R*^2^	0.2183		
*p*-Value	0.0337		
	Estimate	SE	*p* value
Gender (male/female)	0.336	0.171	0.0588
Age, y	0.010	0.015	0.4942
Diabetes mellitus (yes/no)	0.156	0.165	0.3506
Hyperlipidemia (yes/no)	−0.200	0.166	0.2391
CAD (yes/no)	−0.473	0.183	**0.0151**

CAD: Coronary artery disease.
